# Treatment with Raloxifene Induces the Expression of Kisspeptin, Insulin, and Androgen Receptors in Bones of Castrated Adult Female Rats *

**DOI:** 10.1055/s-0044-1779319

**Published:** 2024-04-10

**Authors:** Fernanda Lopes de Freitas Condi, Luiz Fernando Portugal Fuchs, Katia Candido Carvalho, Edmund Chada Baracat

**Affiliations:** 1 Departamento de Ortopedia, Santa Casa de Maringá, Maringá, PR, Brasil; 2 Departamento de Ginecologia, Escola Paulista de Medicina, Universidade Federal de São Paulo, São Paulo, SP, Brasil; 3 Laboratório de Ginecologia Estrutural e Molecular (LIM 58), Disciplina de Ginecologia, Departamento de Obstetrícia e Ginecologia, Hospital das Clínicas, Faculdade de Medicina, Universidade de São Paulo (HCFMUSP), São Paulo, SP, Brasil

**Keywords:** raloxifene hydrochloride, estrogens, genistein, kisspeptins, osteoporosis

## Abstract

**Objective** To evaluate the effects of estrogen, raloxifene and genistein on the expression of *KISS1* (kisspeptin), *KISS1R* (kisspeptin receptor), *AR* (androgen receptor) and *INSR* (insulin receptor) in the bones of ovariectomized rats.

**Methods** Forty-eight adult rats were randomly divided into 6 groups, containing 8 animals each: G1–nonovariectomized control; G2–ovariectomized and treated with conjugated equine estrogens (50 µg/Kg/day); G3–ovariectomized and treated with raloxifene (0.75 mg/kg/day); G4–ovariectomized animal that received soy extract with genistein (300 mg/kg/day); G5–ovariectomized animal that received estrogen and genistein; and G6–ovariectomized animal that received estrogen and raloxifene. Three months after surgery, the castrated animals received the drugs orally daily for 120 days. All animals were sacrificed after this period, by deepening the anesthesia. The left tibia was removed for total RNA extraction and analysis of gene expression of *KISS1* , *KISS1R* , *AR* and *INSR* , by quantitative real-time polymerase chain reaction (qRT-PCR).

**Results** *KISS1* was not detected in any of the treated groups. *KISS1R* , *INSR* and *AR* showed higher expression in the G3 group ( *p* < 0.001), while lower levels of transcripts for these genes were observed in G4 and G5. G2 animals showed hypoexpression of the evaluated genes.

**Conclusion** The results indicate that raloxifene, alone or combined with estrogen, was able to induce the expression of genes associated with the recovery of bone tissue homeostasis in ovariectomized rats.

## Introduction

Osteoporosis presents itself as a global and growing challenge. One in two women, especially postmenopausal women, has this condition. 1 Although age and osteoporosis may be independent factors, 34% of all fractures observed in women are related to aging and a decrease in sex hormone levels. It is known that this reduction leads to an imbalance between bone formation and resorption, with a progressive decrease in mineralization and tissue structure, and consequently an increase in the risk of fractures. 2

Characterized by the loss of bone mass and deterioration of its microarchitecture, 1 2 osteoporosis causes disability, reduced quality of life, and increased mortality due to the high risk of serious fractures. 3 The increasing incidence of osteoporosis, as well as healthcare costs in an aging society, accentuate the need to better understand the physiology and pathogenesis of bone loss. 2 3 In this sense, recent studies highlight the importance of reproductive axis molecules in bone physiology, including kisspeptin, during the remodeling of this tissue.

Kisspeptin is a neuropeptide produced in the hypothalamus and acts in the synthesis of important hormones, such as luteinizing hormone (LH) and follicle-stimulating hormone (FSH). Both kisspeptin and its receptor are essential for reproduction in men and women. 4 It was recently demonstrated in rodents that its administration promotes the *in vitro* differentiation of osteoblasts through the expression of its receptor (KISS1R or GPR54). 4 5 Thus, kisspeptin may have direct beneficial effects on skeletal homeostasis, independently of its role in the release of sex steroids. 5 However, there are few data on its expression and function, as well as its receptor, in bone, especially in osteoporosis related to hypoestrogenism.

Bone tissue samples from affected patients are difficult to obtain, making the use of experimental models an excellent option to help better understand this disease. Thus, the development of an experimental model of ovariectomy has contributed to the investigation of the etiology and pathophysiology of osteoporosis, as well as the establishment of preventive measures and new therapies. 6 Castration of female rats induces a pathological process similar to that of diseases that affect the bone structure, with consequent weight gain and decreased absorption of intestinal calcium. 6 7 The experimental model that best mimics osteoporosis in postmenopausal women is obtained by performing ovariectomy in rats > 6 months old. 7 8

Aware of the relevance of estrogen in physiology and the complications of classical hormonal therapy and selective estrogen receptor modulators (SERMs), phytoestrogens have aroused great clinical interest as a therapeutic alternative. These molecules show high structural and chemical similarity to estrogens. For instance, genistein shares a structure similar to that of 17 β-estradiol, enabling it to bind to estrogen receptors (ERs) and functionally possess estrogenic and antiestrogenic activities. 6 7

Raloxifene (benzothiophene analogue) acts as a selective modulator of estrogen receptors, being used in the treatment and prevention of osteoporosis. Studies show that raloxifene increases bone density and reduces the risk of vertebrae fractures by up to 50% in women with early menopause. 9

In the present study, we conducted a comparative assessment of the effects of treatments involving estrogen, raloxifene, and genistein-rich soy extract, either individually or in combination, on the gene expression patterns of kisspeptin (KISS1) and its receptor (KISS1R) in the bones of ovariectomized rats. Additionally, we examined the expression levels of androgen receptors (ARs) and insulin receptors (INSRs).

## Methods

### Animal treatment and euthanasia

A total of 48 newborn rats ( *Rattus norvegicus albinus* ) of the Wistar lineage were obtained from the central vivarium of our university. The animals were kept in plastic cages measuring 45 × 35 × 15cm, with a metal mesh lid, with food and water ad libitum, at an ambient temperature of 22°C and artificial lighting. A 12-hour light photoperiod was maintained interspersed with a 12-hour dark period, considering the light period from 7:00 am to 7:00 pm. This protocol was evaluated and approved by the Animal Research Ethics Committee under number 0421/07.

**Fig. 1:**
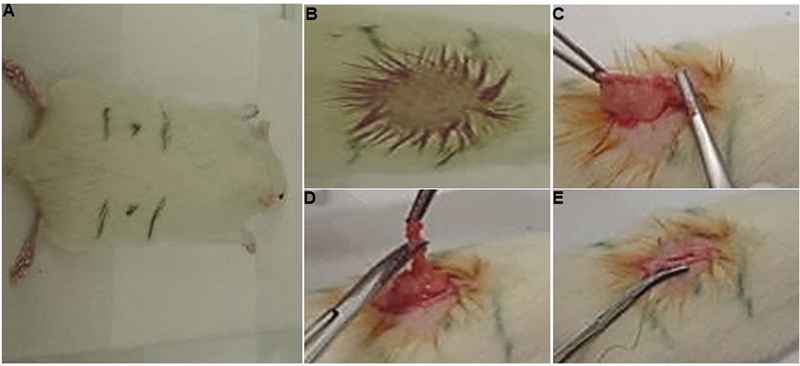
Ovariectomy of adult rats (6 months old). Photographs of the stages of the ovariectomy technique performed in the present study. ( **A** ) Delimitation of the area where the ovary is located; ( **B** ) shaving the back and performing aseptic preparation with Povidine (aqueous solution); ( **C** ) exposure of the left horn of the uterus; ( **D** ) surgical removal of the left ovary; and ( **E** ) closure of the incisions layer by layer.

Nine days after birth, the rats received testosterone propionate (0.1 mg/g), subcutaneously, in order to induce an increase in final bone mass. 6 7 After 6 months of life, the weight range observed was 278 g to 312 g. At that time, 40 rats underwent ovariectomy ( Fig. 1 ). The others, not castrated, constituted the control group (G1). After 21 days, a colpocytological examination was carried out to prove the effect of castration. 6 7

Three months after ovariectomy, the 40 rats were randomly divided into 5 equal groups, containing 8 rats each, namely: G2–ovariectomized rats that received conjugated equine estrogens, dose of 50 µg/Kg/day; G3–ovariectomized rats that received raloxifene, 0.75 mg/kg/day; G4–ovariectomized rats that received soy extract enriched with genistein, 300 mg/kg/day; G5–ovariectomized rats that received soy extract enriched with genistein and combined estrogens; and G6–ovariectomized rats that received raloxifene and combined estrogens. These substances were administered for 120 consecutive days, with the aid of a metal probe, as previously described. 6 7

After the treatment period, the animals were euthanized in a CO2 chamber in accordance with the institutional guideline for animal management of the School of Medicine of Universidade de São Paulo, São Paulo, Brazil. Next, the tibias were collected for histological, immunohistochemical and molecular processing, according to the specific protocol to be described for each analysis.

### Tissue collection and gene expression analysis by real-time quantitative polymerase chain reaction (qRT-PCR)

The right tibia was quickly dissected on a cooled surface (4°C), frozen in liquid nitrogen and stored in a -80°C freezer. To extract total RNA, the tibiae were pulverized in liquid nitrogen using a mortar and steel pestle (Thermo Fisher Scientific Inc., Waltham, MA, United States) previously cooled in dry ice. Trizol reagent (Invitrogen, Thermo Fisher Scientific) was added to the bone powder. This mixture was homogenized with the aid of the Polytron PT10-35 device (Kinematica AG, Malters, Switzerland.

The RNA obtained was treated with DNAse I (Fermentas, Hanover, MD, United States), as recommended by the manufacturer, to eliminate possible contamination with genomic DNA. The concentration and purity of the RNAs were determined by spectrophotometry in a Nano Drop device (Thermo Fisher Scientific) and by electrophoresis in 1% agarose gels. Complementary DNA (cDNA) was synthesized from 1μg of extracted total RNA, using the HiCapacity cDNA synthesis kit (Thermo Fisher Scientific), according to the protocol determined by the manufacturer, on the Veriti device (ThermoFisher Scientific) and standard cycling of the device. The primer oligonucleotides for amplification were designed using Primer Express (Applied Biosystems, Foster City, CA, United States) software, version 1.0. For real-time polymerase chain reaction (RT-PCR), inventoried assays (set of primers and fluorescent probes) were used to amplify the genes *ACTB* (beta-actin, 4352340E), *KISS1* (kisspeptin, Rn00710914_m1), *KISS1R* (receptor kisspeptin, Rn00576940_m1), *INSR* (insulin receptor, Rn00690703_m1) and *AR* (androgen receptor, Rn00560747_m1). The reactions were carried out in triplicate (3 replicates for each gene and samples tested in the analyses), in 96-well plates and using the ABI Prism 7500 apparatus (Applied Biosystems, Foster City, CA, United States).

The values related to the amplification of the genes of interest in the treated groups, in relation to the animals in the control group and normalized by the *ACTB* gene, were obtained using the 2(-delta delta C(T)) (ddCT) method. 10

### Statistical analysis

The data were analyzed in a Microsoft Excel (Microsoft Corporation, Redmond, WA, United States) spreadsheet and the means and standard deviations (SDs) of the mean were calculated for each evaluated group. Initially, the sample distribution was evaluated. For comparison between groups, when the distribution was homogeneous, the analysis of variance (ANOVA) test ( *p* < 0.05) was used, followed by the Tukey post-test. Otherwise, the Kruskal-Wallis test was used, followed by the Dunn test.

To compare two groups, the Student t-test or the Mann-Whitney test were used, depending on the sample distribution. It was considered significant when *p* < 5%. All analyzes were performed using SPSS Statistics for Windows, version 18.0 (SPSS Inc., Chicago, IL, United States).

## Results

**Fig. 2:**
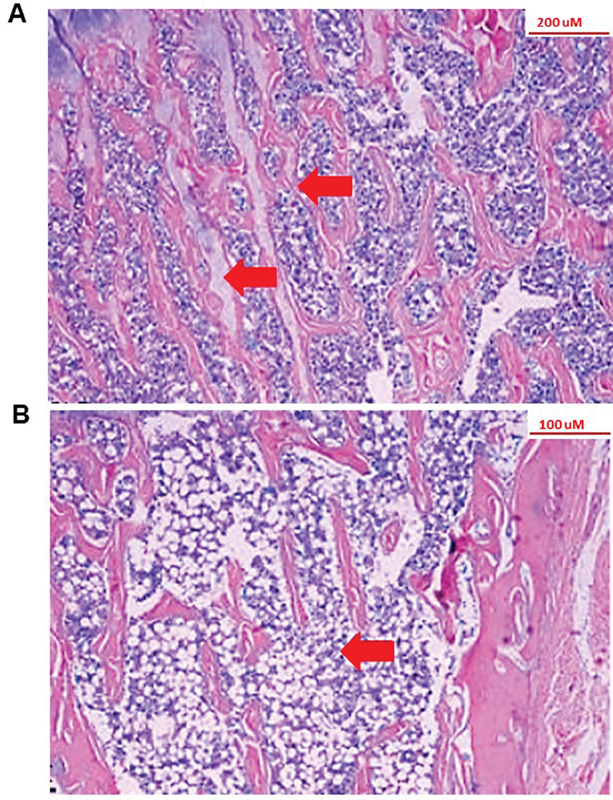
Effect of castration on bone microarchitecture. Bone photomicrograph of nonovariectomized ( **A** ) and ovariectomized ( **B** ) female rats, showing the difference in architecture and tissue organization after castration. Arrows indicate the location of bone trabeculae. The images demonstrate the efficiency of castration in bone tissue.

No statistically significant variation in weight was observed between animals in different groups ( *p* < 0.05), as described in a previous work by our group. 7 These results showed that, after the 4 ^th^ month of treatment, the highest weight was observed for animals in the group treated with estrogen and genistein (G5 = 336.7 g) and the lowest in those treated with raloxifene alone (G3 = 307.6 g). 7 In Fig. 2 , representative photomicrographs of the bone tissue profile (tibia) of noncastrated rats can be seen, compared with the bone tissue of an ovariectomized rat. The figure allows us to observe the difference in the histoarchitecture of bone trabeculae induced by the procedure, demonstrating the effectiveness of the model used in our studies. 7

**Fig. 3:**
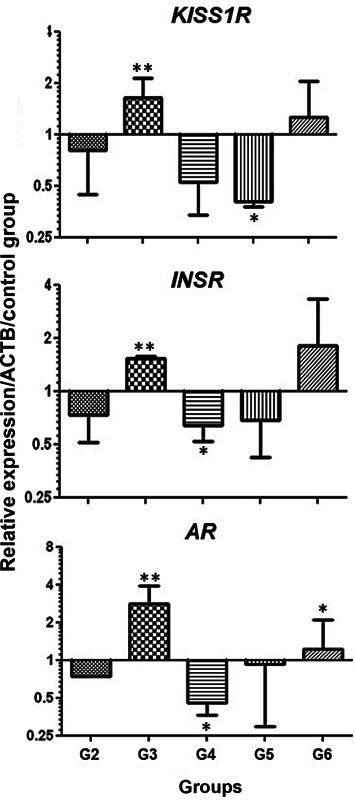
Detection of gene expression by real-time polymerase chain reaction (RT-PCR). Relative expression of KISS1R, INSR and bone AR in rats from the different evaluated groups. Gene expression values were obtained by the ddCT method, using as a reference the amplification values of the nonovariectomized control group, after normalizing the amplification values of each sample (for all target genes) by the values obtained for the constitutive gene ( *ACTB* , beta-actin). Asterisks indicate *p* -values (* *p* < 0.05 and ** *p* < 0.001).

Our group had already demonstrated 6 7 the effectiveness of these treatments in remodeling bone architecture. Therefore, in the present work, we evaluate the influence of these treatments on the expression of genes relevant to reproduction and whose loss has been associated with bone demineralization. The qRT-PCRs showed that greater induction of KISS1R, INSR and AR expression were observed in the group treated with raloxifene (G3), with statistically significant values ( *p* < 0.001). Lower levels of transcripts for these same genes were observed in the G4 and G5 groups (genistein and genistein combined with estrogen, respectively), but without significant differences. Fig. 3 presents the expression values obtained for each group tested.

Animals treated with estrogen alone (G2) showed hypoexpression of all evaluated genes, but without a statistically significant difference in relation to the other groups. The G6 group (treated with raloxifene and estrogen) showed greater expression of the genes evaluated than the groups that received estradiol alone or in combination with genistein, but a significant difference was only observed for AR ( Fig. 3 ).

## Discussion

It is known that, in hypoestrogenism, osteocytes die, with clear recruitment and greater activity of osteoclasts. These, in turn, lead to the degradation of bone tissue and phagocytosis of osteocytes. 11 12 13 Studies have shown osteocytes, osteoblasts and/or bone lining cells, in the process of apoptosis, being phagocytosed by osteoclasts. 12 13 Perhaps this is the main mechanism of action of estrogen on bone.

Estrogen inhibits bone resorption, acting on osteoclasts through several pathways. In hypoestrogenism, there is an increase in the formation and action of osteoclasts, as well as their survival time, inducing a greater number and resorptive activity of these cells. In parallel, the synthesis of bone matrix by osteoblasts is reduced, with greater reabsorption occurring in relation to formation, 14 as well as the disarray in bone microarchitecture seen in our experiment, when comparing ovariectomized animals with the physiological control group. 12 14

Evidence shows that estrogen, raloxifene and genistein-enriched soy extract can attenuate bone changes caused by hypoestrogenism. 6 7 9 Our study showed a positive effect of the association of estrogen and raloxifene on the expression of genes of interest in the bones of castrated rats. However, greater efficacy was observed for raloxifene alone. As for soy extract rich in genistein, isolated or associated with estrogen, no significant beneficial action was observed on bone tissue. Some studies have shown that genistein is not effective in maintaining bone mass after ovariectomy, both in animals and in postmenopausal women. 15

Lower levels of gene expression were observed in the bones of rats treated with estrogen alone. In this sense, the mechanisms of action of this hormone on bones are not completely elucidated, although some of its routes of action are well characterized. It is known that estradiol 17β-benzoate inhibits the formation and activity of osteoclasts, leading to a reduction in the number of these cells. 16 17 This characteristic of estrogen action could justify the loss of gene expression in the groups treated with estradiol in the present study. Faloni et al. 18 observed that the alveolar bones of rats treated with estrogen showed a decrease in the number of osteoclasts due to the induction of apoptosis. Although this data is positive for bone density, the loss in cellularity could compromise the detection of transcripts.

Raloxifene can act as an agonist or antagonist in a tissue-specific manner, as it has an affinity for the ER similar to 17β-estradiol. 19 Some studies suggest that raloxifene is capable of stimulating estrogenic pathways exclusively through ER _β_ , justifying its tissue selectivity. 9 20 21 Our results showed that its isolated action was beneficial to the bone; however, as already described, the association with estrogen did not present an adjuvant effect. This result can be explained by competition for the receptor, or by inhibition of the estrogen receptor with this hormonal combination. 21 22 A previous study demonstrated that raloxifene is more effective in bone tissues with greater estrogen receptor availability. 23

Previous results from our group showed that treatment with genistein and raloxifene increases the expression of type I collagen and its messenger RNA. However, the combined use of conjugated equine estrogens and raloxifene or genistein does not appear to improve or reduce bone quality after ovariectomy. 6 7

Kisspeptin is a peptide encoded by the *KISS1* gene and is important in modulating the hypothalamic-pituitary-gonadal (HHG) axis. It is produced in the hypothalamus, specifically in its anteroventral periventricular area and in the arcuate nucleus, and is the ligand for G protein-linked receptor 54 (GPR54 = KISS1R). In humans, mutations in the gene that encodes KISS1R lead to deficiency in the production of gonadotropin-releasing hormone (GnRH), which culminates in hypogonadotropic hypogonadism. 24 25

The interaction of kisspeptin with its receptor is associated with the GnRH/LH peaks that precede the ovulation hormonal cascade in sheep 26 and rats. 27 In models of androgenized rats with dihydrotestosterone (DHT), a decrease in the expression of the *KISS1* gene and also of its protein was demonstrated, 28 which corroborates our findings. The *KISS1R* gene showed increased expression in groups treated with raloxifene, alone or combined with estradiol, which appears to be a compensatory mechanism for the decreased expression of *KISS1* . In a study carried out by Gore et al., 29 treatment with estradiol from 19 days of fetal life until 7 days of age caused a reduction in the expression of *KISS1* and an increase in *KISS1R* .

The bidirectional influence between bone and energy metabolism was demonstrated by the discovery that the product of osteocalcin (osteoblasts) increases, among other findings, insulin secretion and sensitivity. On the other hand, the anabolic action of insulin is crucial for the function of osteoblasts. This relationship is clear in diabetic patients with severe osteopenia due to insulin deficiency. 30

The present work showed that the treatment of ovariectomized adult rats with raloxifene, alone or combined with estrogen, induced an increase in the expression of *KISS1R* , *INSR* and *AR* . Although studies that define the best conditions for using these drugs, including women with hypoestrogenic osteoporosis, are necessary, our data show that this treatment can be an important alternative in the recovery of bone tissue homeostasis in these patients.

## Conclusion

The results obtained indicate that the use of raloxifene was able to induce the hyperexpression of the *KISS1R* , *INSR* , and *AR* genes in castrated adult female rats.
