# Supplementary material for: Treatment with Raloxifene Induces the Expression of Kisspeptin, Insulin, and Androgen Receptors in Bones of Castrated Adult Female Rats
Source: Rev Bras Ortop (Sao Paulo). 2024 Apr 10;59(2):e228–34. doi: 10.1055/s-0044-1779319 (PMC11006519; doi:10.1055/s-0044-1779319)
Supplement: Related texts

# Research article: Tratamento com raloxifeno induz à expressão dos receptores de kisspeptina, insulina e androgênio em ossos de ratas adultas castradas *

**Keywords:** cloridrato de raloxifeno, estrogênio, genisteína, kisspeptinas, osteoporose

## Abstract

**Objetivo** Avaliar os efeitos do estrogênio, raloxifeno e genisteína na expressão de *KISS1* (kisspeptina), *KISS1R* (receptor da kisspeptina), *AR* (receptor de androgênio) e *INSR* (receptor de insulina) nos ossos de ratas ovariectomizadas.

**Métodos** Quarenta e oito ratas adultas foram divididas aleatoriamente em 6 grupos, contendo 8 animais cada: G1–controle não ovariectomizado); G2–ovariectomizado e tratado com estrogênios conjugados equinos (50 µg/Kg/dia); G3–ovariectomizado e tratado com raloxifeno (0,75 mg/kg/dia); G4–ovariectomizado que recebeu extrato de soja com genisteína (300 mg/kg/dia); G5–ovariectomizado que recebeu estrogênio e genisteína; e G6–ovariectomizado que recebeu estrogênio e raloxifeno. Após 3 meses da cirurgia, os animais castrados receberam os fármacos diariamente por via oral, durante 120 dias. Todos os animais foram sacrificados após esse período, por aprofundamento da anestesia. A tíbia esquerda foi removida para extração de RNA total e análise da expressão gênica de *KISS1, KISS1R, AR* e *INSR,* por reação de cadeia de polimerase quantitativa em tempo real ( *quantitative real-time polymerase chain reaction* , qRT-PCR, em inglês).

**Resultados** *KISS1* não foi detectado em nenhum dos grupos tratados. *KISS1R* , *INSR* e *AR* mostraram maior expressão no grupo G3 ( *p* < 0,001), enquanto menores níveis de transcritos para esses genes foram observados em G4 e G5. Os animais de G2 apresentaram hipoexpressão dos genes avaliados.

**Conclusão** Os resultados indicam que o raloxifeno, isolado ou combinado com estrogênio, foi capaz de induzir a expressão de genes associados à recuperação da homeostase do tecido ósseo em ratas ovariectomizadas.

## Introdução

A osteoporose apresenta-se como um desafio global e crescente. Uma em cada duas mulheres, principalmente na pós-menopausa, apresenta esta afecção. 1 Embora a idade e a osteoporose possam ser fatores independentes, 34% do total de fraturas observadas nas mulheres estão relacionadas ao envelhecimento e à diminuição nos níveis dos hormônios sexuais. Sabe-se que esta queda leva ao desequilíbrio entre a formação e reabsorção ósseas, com progressiva redução da mineralização e estrutura do tecido, e consequentemente ao aumento do risco de fraturas. 2

Caracterizada pela perda da massa óssea e deterioração de sua microarquitetura, 1 2 a osteoporose causa incapacidade, redução da qualidade de vida e aumento da mortalidade devido ao risco elevado de fraturas graves. 3 O aumento na incidência da osteoporose, bem como os custos de saúde em uma sociedade envelhecida, acentuam a necessidade de entender melhor a fisiologia e a patogênese da perda óssea. 2 3 Neste sentido, estudos recentes evidenciam a importância de moléculas do eixo reprodutivo na fisiologia óssea, entre eles a kisspeptina, durante a remodelação deste tecido.

A kisspeptina é um neuropeptídeo produzido no hipotálamo e atua na síntese de importantes hormônios, como o hormônio luteinizante ( *luteinizing hormone* , LH, em inglês) e o hormônio folículo estimulante ( *follicle-stimulating hormone* , FSH, em inglês). Tanto a kisspeptina quanto seu receptor são essenciais para a reprodução em homens e mulheres. 4 Foi demonstrado recentemente em roedores que sua administração promove a diferenciação *in vitro* de osteoblastos pela expressão do seu receptor ( *KISS1R* ou *GPR54* ). 4 5 Assim, a kisspeptina pode ter efeitos benéficos diretos na homeostase esquelética, independentemente da sua atuação na liberação de esteroides sexuais. 5 No entanto, existem poucos dados sobre sua expressão e função, bem como de seu receptor, no osso, principalmente na osteoporose relacionada ao hipoestrogenismo.

Amostras de tecido ósseo de pacientes acometidas são de difícil obtenção, tornando a utilização de modelos experimentais uma excelente opção para auxiliar a melhor compreender esta doença. Assim, o desenvolvimento de modelo experimental de ovariectomia tem contribuído na investigação da etiologia e patofisiologia da osteoporose, bem como no estabelecimento de medidas preventivas e novas terapias. 6 A castração das ratas induz a um processo patológico similar ao de doenças que afetam a estrutura óssea, com consequente ganho de peso e diminuição da absorção do cálcio intestinal. 6 7 O modelo experimental que melhor mimetiza a osteoporose da mulher na pós-menopausa é obtido com a realização da ovariectomia em ratas com mais de seis meses de idade. 7 8

Cientes da relevância do estrogênio na fisiologia e das complicações da terapia hormonal clássica e dos moduladores seletivos do receptor estrogênico ( *selective estrogen receptor modulators* , SERMs, em inglês), os fitoestrogênios despertaram grande interesse clínico como alternativa terapêutica. Estas moléculas apresentam alta similaridade estrutural e química com os estrogênios. A genisteína, por exemplo, tem sua estrutura semelhante à do 17 β-estradiol, podendo se ligar aos receptores de estrogênio (REs), tendo funcionalmente atividade estrogênica e antiestrogênica. 6 7

O raloxifeno (análogo do benzotiofeno) atua como modulador seletivo dos receptores de estrogênio, sendo usado no tratamento e prevenção da osteoporose. Estudos mostram que o raloxifeno aumenta a densidade óssea e reduz em até 50% o risco de fraturas de vértebras em mulheres com menopausa precoce. 9

No presente trabalho, avaliamos comparativamente os efeitos dos tratamentos com estrogênio, raloxifeno e extrato de soja rico em genisteína, isolados ou em combinação, no perfil de expressão gênica da kisspeptina ( *KISS1* ) e de seu receptor ( *KISS1R* ) no osso de ratas ovariectomizadas. De igual modo, a expressão dos receptores de androgênio ( *AR* ) e de insulina ( *INSR* ) também foram analisados.

## Métodos

## Tratamento dos animais e eutanásia

Ao todo, 48 ratas ( *Rattus norvegicus albinus* ) recém-nascidas, da linhagem Wistar, foram obtidas no biotério central da nossa universidade. Os animais foram mantidos em gaiolas plásticas medindo 45 × 35 × 15 cm, com tampa gradeada de metal, com alimentação e água *ad libitum,* em temperatura ambiente de 22°C e iluminação artificial. Manteve-se fotoperíodo claro de 12 horas intercalado com escuro de 12 horas, considerando-se o período de luz das 7:00 às 19:00 horas. O presente protocolo foi avaliado e aprovado pela Comissão de Ética no Uso de Animais (CEUA; n° 0421/07).

Após 9 dias de nascimento, as ratas receberam propionato de testosterona (0,1 mg/g), pela via subcutânea, a fim de induzir aumento da massa óssea final. 6 7 Após os 6 meses de vida, a faixa de peso observada foi de 278 g a 312 g. Neste momento, 40 ratas foram submetidas à ovariectomia ( Fig. 1 ). As demais, não castradas, constituíram o grupo controle (G1). Após 21 dias, foi realizado exame colpocitológico para comprovar o efeito da castração. 6 7

**Fig. 1:** Ovariectomia das ratas adultas (6 meses de idade). Fotografias as etapas da técnica de ovariectomia realizadas no presente estudo. ( **A** ) Delimitação da área onde se encontra o ovário; ( **B** ) depilação do dorso e assepsia com Povidine (solução aquosa); ( **C** ) exposição do corno esquerdo do útero; ( **D** ) exérese do ovário esquerdo; e ( **E** ) fechamento por planos.

Após 3 meses da ovariectomia, as 40 ratas foram divididas randomicamente em 5 grupos iguais, contendo 8 ratas cada, a saber: G2–ratas ovariectomizadas que receberam estrogênios conjugados equinos, dose de 50 µg/Kg/dia; G3–ratas ovariectomizadas que receberam raloxifeno, 0,75 mg/kg/dia; G4–ratas ovariectomizadas que receberam extrato de soja enriquecido com genisteína, 300 mg/kg/dia; G5–ratas ovariectomizadas e que receberam extrato de soja enriquecido com genisteína e estrogênios combinados e G6–ratas ovariectomizadas que receberam raloxifeno e estrogênios combinados. Estas substâncias foram ministradas durante 120 dias consecutivos, com auxílio de sonda metálica, como previamente descrito. 6 7

Após o período de tratamento, os animais foram submetidos a eutanásia em câmara de CO _2_ conforme a diretriz institucional para manejo de animais da Faculdade de Medicina da Universidade de São Paulo. Em seguida, as tíbias foram coletadas para processamento histológico, imunoistoquímico e molecular, conforme protocolo específico a ser descrito para cada análise.

## Coleta dos tecidos e análise da expressão gênica por reação de cadeia de polimerase quantitativa em tempo real

A tíbia direita foi dissecada rapidamente sobre uma superfície resfriada (4°C), congelada em nitrogênio líquido e armazenada em freezer -80 °C. Para a extração do RNA total, as tíbias foram pulverizadas, em nitrogênio líquido, utilizando um mortar e pistilo de aço (Thermo Fisher Scientific, Inc., Waltham, MA, Estados Unidos) previamente resfriados em gelo seco. Ao pó de osso, foi adicionado o reagente Trizol (Invitrogen, Thermo Fisher Scientific). Essa mistura foi homogeneizada com auxílio do aparelho Polytron PT10-35 (Kinematica AG, Malters, Suíça).

O RNA obtido foi tratado com *DNAse I* (Fermentas, Hanover, MD, Estados Unidos), segundo indicação do fabricante, para eliminar possível contaminação com DNA genômico. A concentração e pureza dos RNAs foram determinadas por espectrofotometria em aparelho tipo Nano Drop (Thermo Fisher Scientific) e por eletroforese em géis de agarose a 1%. O DNA complementar (cDNA) foi sintetizado a partir de 1μg do RNA total extraído, utilizando o kit HiCapacity cDNA synthesis (Thermo Fisher Scientific), conforme protocolo determinado pelo fabricante, no aparelho Veriti (ThermoFisher Scientific) e ciclagem padrão do aparelho. Os oligonucleotídeos iniciadores para a amplificação foram desenhados com auxílio do programa Primer Express (Applied Biosystems, Foster City, CA, Estados Unidos), versão 1.0. Para as reações de reação em cadeia da polimerase em tempo real ( *real-time polymerase chain reaction* , RT-PCR, em inglês) foram utilizados *assays* inventoriados (conjunto de *primers* e sondas fluorescentes) para amplificação dos genes *ACTB* (beta-actina, 4352340E), *KISS1* (kisspeptina, Rn00710914_m1), *KISS1R* (receptor da kisspeptina, Rn00576940_m1), *INSR* (receptor de insulina, Rn00690703_m1) e *AR* (receptor de androgênio, Rn00560747_m1). As reações foram realizadas em triplicada (três réplicas para cada gene e amostras testada nas análises), em placas de 96 poços e utilizando o aparelho ABI Prism 7500 (Applied Biosystems, Foster City, CA, Estados Unidos).

Os valores relativos à amplificação dos genes de interesse nos grupos tratados em relação aos animais do grupo controle e normalizados pelo gene *ACTB* foram obtidos pelo método 2(-delta delta C(T)) (ddCT). 10

## Análise Estatística

Os dados foram analisados em planilha do Microsoft Excel (Microsoft Corporation, Redmond, WA, Estados Unidos) e foram calculados as médias e os desvios padrão (DPs) da média para cada grupo avaliado. Inicialmente, avaliou-se a distribuição da amostra. Para a comparação entre os grupos, quando a distribuição foi homogênea, utilizou-se o teste de análise de variância ( *analysis of variance* , ANOVA, em inglês) ( *p* < 0,05), seguido pelo pós-teste de Tukey. Caso contrário, foi empregado o teste de Kruskal-Wallis, seguido pelo teste de Dunn.

Para a comparação entre dois grupos, foram empregados os testes t de Student ou Mann-Whitney, na dependência da distribuição da amostra. Fixou-se como significante quando *p* < 5%. Todas as análises foram realizadas com o software estatístico SPSS Statistics for Windows, versão 18.0 (SPSS Inc., Chicago, IL, Estados Unidos).

## Resultados

Não foi observada variação estatisticamente significativa de peso entre os animais dos diferentes grupos ( *p* < 0,05), conforme descrito em trabalho anterior de nosso grupo. 7 Estes resultados mostraram que, após o 4° mês de tratamento, maior peso foi observado para os animais do grupo tratado com estrogênio e genisteína (G5 = 336,7 g) e o menor naqueles tratados apenas com raloxifeno (G3 = 307,6 g). 7 Na Fig. 2 , podem ser observadas as fotomicrografias representativas do perfil de tecido ósseo (tíbia) de ratas não castradas, em comparação ao tecido ósseo de uma rata ovariectomizada. A figura permite observar a diferença na histoarquitetura das trabéculas ósseas induzida pelo procedimento, demonstrando a eficácia do modelo utilizado em nossos estudos. 7

**Fig. 2:** Efeito da castração na microarquitetura óssea. Fotomicrografia óssea de ratas não ovariectomizada ( **A** ) e ovariectomizada ( **B** ), apresentando a diferença da arquitetura e organização do tecido após a castração. As setas indicam a localização das trabéculas ósseas. As imagens demonstram a eficiência da castração no tecido ósseo.

Nosso grupo já havia demonstrado 6 7 a eficácia destes tratamentos na remodelação da arquitetura óssea. Assim, no presente manuscrito, avaliamos a influência destes tratamentos na expressão de genes relevantes para a reprodução e cuja perda tem sido associada à desmineralização óssea. As qRT-PCRs mostraram que maior indução da expressão de *KISS1R* , *INSR* e *AR* foram observadas no grupo tratado com raloxifeno (G3), com valores estatisticamente significativos ( *p* < 0,001). Menores níveis de transcritos para estes mesmos genes foram observados nos grupos G4 e G5 (genisteína e genisteína combinada com estrogênio, respectivamente), porém sem diferença significativa. A Fig. 3 apresenta os valores de expressão obtidos para cada grupo testado.

**Fig. 3:** Detecção da expressão gênica por reação em cadeia da polimerase em tempo real ( *real-time polymerase chain reaction* , RT-PCR, em inglês). Expressão relativa de *KISS1R, INSR* e *AR* óssea nas ratas dos diferentes grupos avaliados. Os valores de expressão gênica foram obtidos pelo método ddCT, utilizando como referência os valores de amplificação do grupo controle não ovariectomizado, após normalização dos valores de amplificação de cada amostra (para todos os genes alvo) pelos valores obtidos para o gene constitutivo ( *ACTB* , beta-actina). Os asteriscos indicam os valores- *p* (* *p* < 0,05 e ** *p* < 0,001).

Os animais tratados somente com estrogênio (G2) apresentaram hipoexpressão de todos os genes avaliados, porém sem diferença estatisticamente significativa em relação aos demais grupos. Já o grupo G6 (tratado com raloxifeno e estrogênio) mostrou maior expressão dos genes avaliados do que os grupos que receberam estradiol isolado ou em combinação com genisteína, porém diferença significativa só foi observada para o *AR* ( Fig. 3 ).

## Discussão

Sabe-se que no hipoestrogenismo ocorre a morte dos osteócitos, com nítido recrutamento e maior atividade dos osteoclastos. Estes, por sua vez, levam à degradação do tecido ósseo e fagocitose dos osteócitos. 11 12 13 Estudos mostraram os osteócitos, osteoblastos e/ou células de revestimento ósseo, em processo de apoptose, sendo fagocitados pelos osteoclastos. 12 13 Talvez este seja o principal mecanismo de ação do estrogênio no osso.

O estrogênio inibe a reabsorção óssea, agindo sobre os osteoclastos por diversas vias. No hipoestrogenismo, ocorre aumento na formação e ação de osteoclastos, bem como de seu tempo de sobrevida, induzindo a maiores número e atividade reabsortiva destas células. Em paralelo, a síntese de matriz óssea pelos osteoblastos é diminuída, ocorrendo maior reabsorção em relação à formação, 14 bem como o desarranjo na microarquitetura óssea visto em nosso experimento, ao compararmos os animais ovariectomizados com o controle fisiológico. 12 14

Evidências mostram que o estrogênio, o raloxifeno e o extrato de soja enriquecido com genisteína podem atenuar as alterações ósseas causadas pelo hipoestrogenismo. 6 7 9 Nosso estudo mostrou efeito positivo da associação de estrogênio e raloxifeno na expressão dos genes de interesse nos ossos das ratas castradas. Contudo, maior eficácia foi observada para o raloxifeno isoladamente. Já para o extrato de soja rico em genisteína, isolado ou associado ao estrogênio, não foi observada ação benéfica significativa no tecido ósseo. Alguns estudos mostraram que a genisteína não é eficaz na manutenção da massa óssea após a ovariectomia, tanto em animais quanto em mulheres na pós-menopausa. 15

Menores níveis de expressão gênica foram observados nos ossos das ratas tratadas somente com estrogênio. Neste sentido, os mecanismos de ação deste hormônio nos ossos não estão completamente elucidados, apesar de algumas de suas vias de ação serem bem caracterizadas. Sabe-se que o 17β-benzoato de estradiol inibe a formação e a atividade dos osteoclastos, levando à redução do número destas células. 16 17 Esta característica de ação do estrogênio poderia justificar a perda de expressão gênica nos grupos tratados com estradiol, no presente estudo. Faloni et al., 18 observaram que os ossos alveolares de ratas tratadas com estrogênio apresentam diminuição do número de osteoclastos, por indução da apoptose. Embora este dado seja positivo para a densidade óssea, a perda na celularidade poderia comprometer a detecção de transcritos.

Já o raloxifeno pode agir como agonista ou antagonista de forma tecido específica, por apresentar afinidade pelo RE semelhante ao 17β-estradiol. 19 Alguns estudos sugerem que o raloxifeno é capaz de estimular as vias estrogênicas exclusivamente pelo RE _β_ , justificando sua seletividade tecidual. 9 20 21 Nossos resultados mostraram que sua ação isolada foi benéfica ao osso; porém, como já descrito, a associação ao estrogênio não apresentou efeito adjuvante. Este resultado pode ser explicado pela competição ao receptor, ou por inibição do receptor de estrogênio com essa combinação hormonal. 21 22 Estudo anterior demonstrou que o raloxifeno é mais eficaz em tecidos ósseos com maior disponibilidade de receptor de estrogênio. 23

Resultados prévios de nosso grupo mostraram que o tratamento com genisteína e o raloxifeno aumentam a expressão de colágeno tipo I e seu RNA mensageiro. No entanto, o uso combinado de estrogênios conjugados equinos e raloxifeno ou genisteína não parecem melhorar ou reduzir a qualidade óssea após ovariectomia. 6 7

A kisspeptina é um peptídeo codificado pelo gene *KISS1* e é importante na modulação do eixo hipotálamo-hipósife-gonadal (HHG). É produzida no hipotálamo, especificamente na sua área ânteroventral periventricular e no núcleo arqueado, e é o ligante do receptor 54 ligado à proteína G ( *GPR54* = *KISS1R* ). Em humanos, mutações no gene que codifica o KISS1R levam à deficiência da produção de hormônio liberador de gonadotrofinas ( *gonadotropin-releasing hormone* , GnRH, em inglês), o que culmina com o quadro de hipogonadismo hipogonadotrófico. 24 25

A interação da kisspeptina com o seu receptor é associada com os picos de GnRH/LH que precedem a cascata hormonal da ovulação em ovelhas 26 e em ratos. 27 Assim, a não detecção do gene *KISS1* em nossos experimentos pode se dever ao estado menopausal dos animais. Em modelos de ratas androgenizadas com DHT (di-hidrotestosterona), foi demonstrada a diminuição da expressão do gene *KISS1* e também de sua proteína, 28 o que corrobora com os nossos achados. Já o gene *KISS1R* apresentou expressão aumentada nos grupos tratados com raloxifeno, isolado ou combinado ao estradiol, o que parece ser um mecanismo compensatório para a expressão diminuída do *KISS1* . Em estudo realizado por Gore et al., 29 o tratamento com estradiol a partir de 19 dias de vida fetal até 7 dias de idade ocasionou uma redução na expressão do *KISS1* e aumento de *KISS1R.*

A influência bidirecional entre osso e metabolismo energético foi demonstrada pela descoberta de que o produto da osteocalcina (osteoblastos) aumenta, entre outros achados, a secreção e sensibilidade à insulina. Em contrapartida, a ação anabólica da insulina mostra-se crucial para a função dos osteoblastos. Essa relação é nítida em pacientes diabéticos com osteopenia grave devido à deficiência de insulina. 30

O presente trabalho mostrou que o tratamento de ratas adultas ovariectomizadas com raloxifeno, isolado ou combinado com estrogênio, induziu aumento da expressão de *KISS1R* , *INSR* e *AR* . Embora estudos que definam as melhores condições de uso desses fármacos, incluindo mulheres com osteosporose hipoestrogênica, sejam necessários, nossos dados mostram que este tratamento pode ser uma importante alternativa na recuperação da homeostase do tecido ósseo destas pacientes.

## Conclusão

Os resultados obtidos indicam que a utilização do raloxifeno foi capaz de induzir a hiperexpressão dos genes *KISS1R* , *INSR* e *AR* em ratas adultas castradas.
